# Correlation of Nav1.8 and Nav1.9 sodium channel expression with neuropathic pain in human subjects with lingual nerve neuromas

**DOI:** 10.1186/1744-8069-9-52

**Published:** 2013-10-21

**Authors:** Emma V Bird, Claire R Christmas, Alison R Loescher, Keith G Smith, Peter P Robinson, Joel A Black, Stephen G Waxman, Fiona M Boissonade

**Affiliations:** 1 Academic Unit of Oral and Maxillofacial Medicine and Surgery, School of Clinical Dentistry, University of Sheffield, Claremont Crescent, Sheffield S10 2TA, UK; 2 Department of Neurology and Center for Neuroscience and Regeneration Research, Yale University School of Medicine, New Haven, CT, USA; 3 Rehabilitation Research Center, VA Connecticut Healthcare System, West Haven, CT, USA

**Keywords:** Lingual nerve, Nerve injury, Trigeminal, Neuropathic pain, Dysaesthesia, Tingling, Nav1.8, Nav1.9

## Abstract

**Background:**

Voltage-gated sodium channels Nav1.8 and Nav1.9 are expressed preferentially in small diameter sensory neurons, and are thought to play a role in the generation of ectopic activity in neuronal cell bodies and/or their axons following peripheral nerve injury. The expression of Nav1.8 and Nav1.9 has been quantified in human lingual nerves that have been previously injured inadvertently during lower third molar removal, and any correlation between the expression of these ion channels and the presence or absence of dysaesthesia investigated.

**Results:**

Immunohistochemical processing and quantitative image analysis revealed that Nav1.8 and Nav1.9 were expressed in human lingual nerve neuromas from patients with or without symptoms of dysaesthesia. The level of Nav1.8 expression was significantly higher in patients reporting pain compared with no pain, and a significant positive correlation was observed between levels of Nav1.8 expression and VAS scores for the symptom of tingling. No significant differences were recorded in the level of expression of Nav1.9 between patients with or without pain.

**Conclusions:**

These results demonstrate that Nav1.8 and Nav1.9 are present in human lingual nerve neuromas, with significant correlations between the level of expression of Nav1.8 and symptoms of pain. These data provide further evidence that changes in expression of Nav1.8 are important in the development and/or maintenance of nerve injury-induced pain, and suggest that Nav1.8 may be a potential therapeutic target.

## Background

The mechanisms underlying the development of chronic pain following peripheral nerve injury are still poorly understood, and neuropathic pain remains a substantial clinical problem. Injury to a peripheral nerve often leads to the development of a neuroma, a disordered mass of axons embedded within the connective and scar tissue of a damaged nerve trunk, and this site has been shown to generate abnormal and spontaneous discharge [1-3]. Within sensory neurons, voltage-gated sodium channels (VGSCs) are responsible for the initiation and generation of action potentials. There is a growing body of evidence that changes in the transcriptional regulation of VGSCs plays a pivotal role in the generation and maintenance of ectopic activity from injured or spared axons at the site of a nerve injury, with a number of subtypes being of particular significance [4-8]. Of the nine distinct voltage-gated sodium channel subtypes identified in mammals, the physiological properties and patterns of expression within the nervous system, of subtypes Nav1.7, 1.8 and 1.9, suggests they may play important role(s) in the initiation and/or maintenance of chronic pain [9-11]. Evidence indicates that Nav1.7, 1.8 and 1.9 are preferentially expressed in nociceptive fibres of the peripheral nervous system, and they have been shown by a number of studies to be predominantly localised in the pain-conducting small and medium-sized neurons of the dorsal root and trigeminal ganglia [12-17]. However there is also evidence that Nav1.8 is expressed in a large proportion of DRG neurons including myelinated A fibres [18]. A number of studies have indicated a role for Nav1.7 in chronic pain in humans [11,19-21]; however, a recent immunohistochemical study in our laboratory reported that although the sodium channel subtype Nav1.7 was expressed in human lingual nerve neuromas, there was no direct relationship between the level of expression and the patients’ symptoms of dysaesthesia [22]. Therefore further investigation into the role that sodium channel subtypes Nav1.8 and 1.9 play in the propagation and initiation of chronic painful symptoms such as dysaesthesia will further our understanding of the underlying mechanisms and may identify useful targets for novel analgesics.

Peripheral nerves in the orofacial region are susceptible to injury during routine oral surgical procedures. The lingual nerve, a sensory branch of the mandibular division of the trigeminal nerve, is susceptible to injury during routine oral surgical procedures or as a consequence of facial trauma. Its anatomical position makes it vulnerable to damage during the surgical extraction of lower third molar teeth, and patients who sustain such an injury are often left with a loss of sensation to the ipsilateral side of their tongue. Furthermore a significant proportion of these patients go on to develop abnormal and unpleasant persistent sensory disturbances such as dysaesthesia, which can be either spontaneous or induced by movement or touching of the tongue [23,24]. There are currently no satisfactory methods for the treatment of dysaesthesia and patients who have sustained a lingual nerve injury, and show little or no spontaneous recovery, are referred for microsurgical repair of the damaged nerve. This has resulted in the collection of an extensive archive of lingual nerve neuromas [25].

With the aim of investigating the role that sodium channel subtypes play in chronic pain arising as a result of lingual nerve injury, the expression of Nav1.8 and Nav1.9 within nerve tissue (as identified by the general neuronal marker PGP9.5) was examined and quantified in human lingual nerve neuromas, and any association between the expression of these sodium channels and the presence or absence of symptoms of dysaesthesia identified. Data from this study show that Nav1.8 and Nav1.9 are expressed in human lingual nerve neuromas, with a significant increase in levels of Nav1.8 expression seen in neuromas taken from patients with symptoms of dysaesthesia. Furthermore a positive correlation of the expression of Nav1.8 with the symptom and degree of tingling experienced by the patients was recorded.

## Results

**Figure 1:**
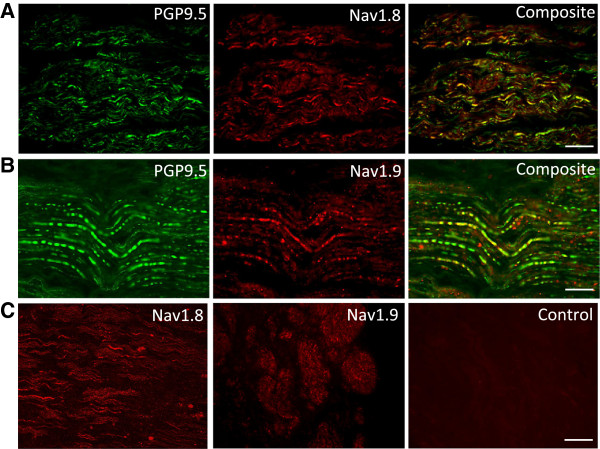
**Nav1.8 and Nav1.9 expression in lingual neuromas. A** and **B.** Nav1.8 and Nav1.9 immunolabelling was bright and appeared to be axon-specific, showing positive co-localisation with PGP9.5. Images showing immunofluorescent labelling in the lingual nerve neuromas for PGP9.5 (green) and Nav1.8 and Nav1.9 (red). The composite images show the expression of Nav1.8 and Nav1.9 within PGP9.5 labelled tissue (yellow). **C.** No positive staining for sodium channel subtypes was present where the primary antibodies were omitted. Images of lingual nerve neuromas following incubation with Nav1.8 antibody, Nav1.9 antibody, and secondary antibody alone (Control). Scale bar = 100 μm **(A and C)**; 50 μm **(B)**.

Of the thirteen specimens selected for analysis, nine were taken from female patients, and four from male patients, with the mean age (±SEM) at the time of microsurgical repair of their lingual nerves being 31.2 ± 1.76 years (range 22–46 years). No labelling was present in tissue sections where primary antibodies for PGP9.5 and the sodium channel subtypes were omitted, as previously described for PGP9.5 [22] and as shown in Figure 1.

### Qualitative and quantitative expression of Nav1.8 in PGP9.5-labelled lingual neuroma tissue

**Figure 2:**
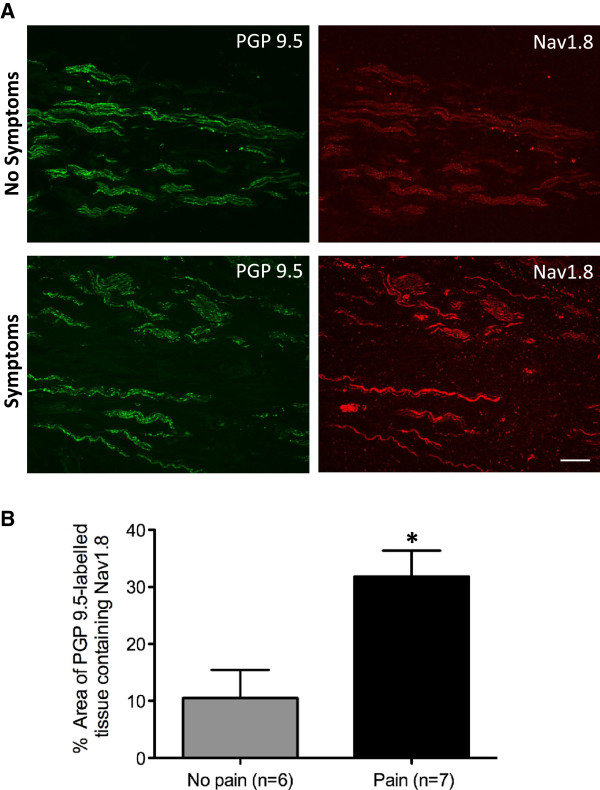
**Nav1.8 is expressed in human lingual nerve neuromas. A.** Nav1.8 immunolabelling appeared greater in lingual nerve neuromas from patients with symptoms of dysaesthesia. Images of lingual nerve neuromas from patients without or with symptoms of dysaesthesia, showing double labelling with the general neuronal marker PGP9.5 (green) and Nav1.8 (red). Scale bar, 100 μm. **B.** The mean percentage area (± SEM) of PGP9.5 labelled tissue containing Nav1.8 was significantly higher in lingual nerve neuroma specimens taken from patients with symptoms of pain (unpaired *t*-test p = 0.0087; no pain 10.52 ± 4.93%, pain 31.82 ± 4.54%).

Immunoreactivity to the general neuronal marker PGP9.5, and Nav1.8 was present in all of the neuromas analysed; however, the level and pattern of expression varied between the individual specimens (Table 1). Some neuromas expressed high levels of Nav1.8 immunolabelling that was bright and appeared to be axonal-specific, showing positive co-localisation with PGP9.5. In contrast, other neuromas exhibited a very low level of Nav1.8 immunoreactivity. However, overall, the level of Nav1.8 expression appeared to be higher in neuroma specimens from patients with symptoms of dysaesthesia compared with that in specimens from patients with no symptoms (Figure 2).

**Table 1:** Details of the patients included in the study, including VAS scores, gender, age of the patients, time between the initial injury and surgical repair, and the Nav1.8 and Nav1.9 expression levels

**No symptoms of dysaesthesia**								
Neuroma specimen	% Nav1.8	% Nav1.9	Gender (male/female)	Age (years)	Pain VAS	Tingling VAS	Discomfort VAS	Time between injury and repair (months)
1	10.62	6.86	F	31	0	0	44	18
2	33.06	5.42	F	25	1	2	10	21
3	12.54	11.87	F	28	1	0	81	16
4	0.21	8.25	F	39	1	2	39	26
5	5.11	-	F	31	1	1	48	11
6	1.58	13.39	M	28	3	1	7	6
**Symptoms of dysaesthesia**								
Neuroma specimen	% Nav1.8	% Nav1.9	Gender (male/female)	Age (years)	Pain VAS	Tingling VAS	Discomfort VAS	Time between injury and repair (months)
7	38.55	9.64	M	28	65	81	50	41
8	54.83	16.33	F	36	29	97	70	14
9	20.19	18.97	F	22	34	99	42	15
10	32.68	14.15	M	27	66	8	50	7
11	29.77	1.12	F	34	79	95	95	9
12	21.42	10.72	F	31	81	50	99	53
13	25.32	18.67	M	46	78	77	75	288

Quantitative analysis of the neuromas labelled for PGP9.5 and Nav1.8 revealed that the fraction of PGP9.5 labelled tissue expressing Nav1.8 ranged from 0.21% to 54.83% (Table 1). In neuromas taken from patients experiencing no symptoms of dysaesthesia, Nav1.8 levels ranged from 0.21% to 33.06%, and in neuromas from patients who did experience painful dysaesthesia, fractional expression of Nav1.8 ranged from 20.19% to 54.83%. Further statistical analysis confirmed the above qualitative observation and demonstrated that there was a statistical significant difference in Nav1.8 expression between the patients reporting symptoms of dysaesthesia compared with those reporting no symptoms (unpaired *t*-test p = 0.0087; pain, 31.82 ± 4.54 [SEM]%, no pain 10.52 ± 4.93 [SEM]%) (Figure 2).

**Figure 3:**
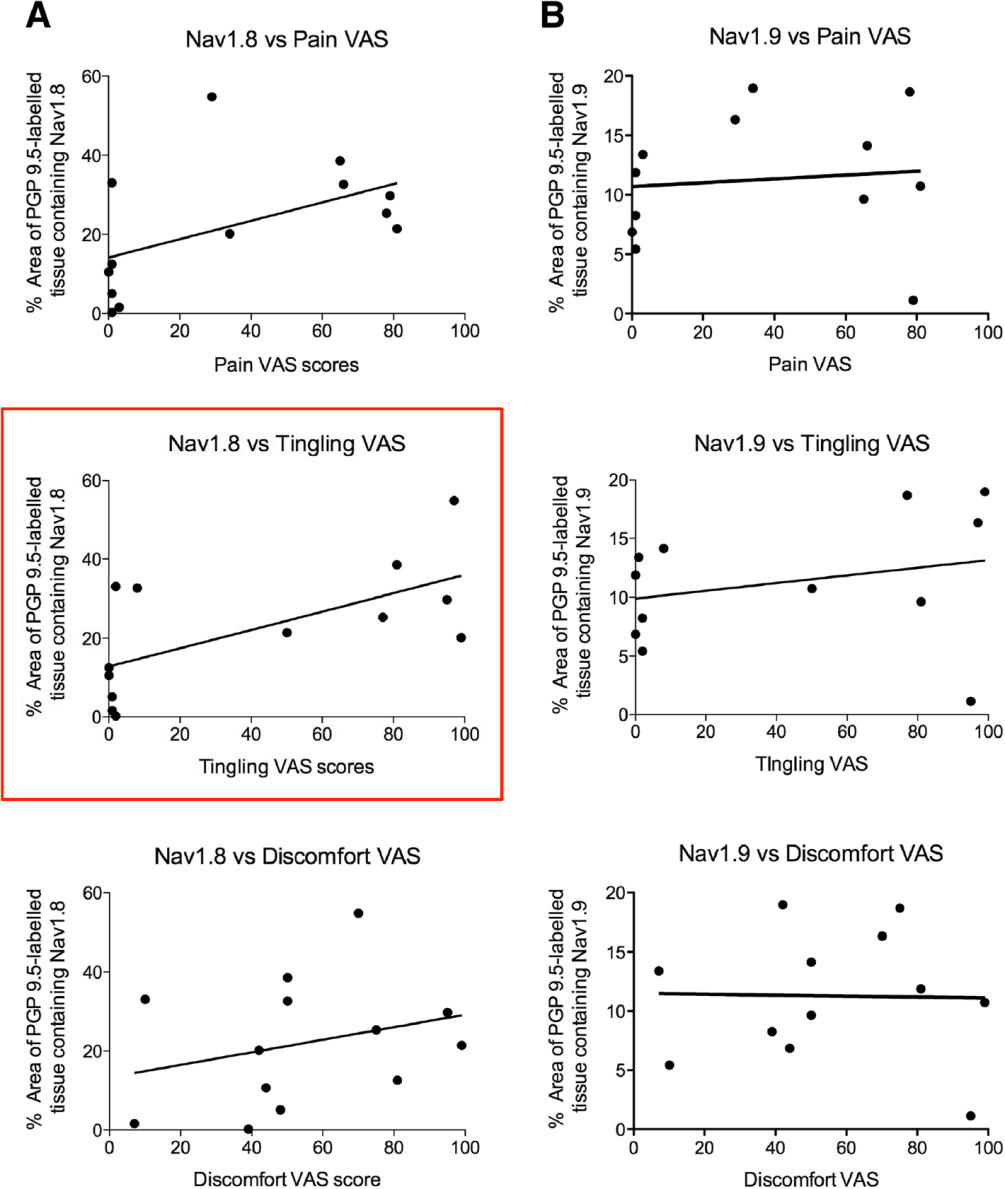
**Correlation of Nav1.8 and Nav1.9 with symptoms of pain, tingling and discomfort. A.** Regression plots showing the relationship between the expression of Nav1.8 and VAS scores for pain, tingling and discomfort as reported by the patient. The level of Nav1.8 expression correlated positively with reported levels of tingling (Pearson’s Correlation *r* = 0.64, *p* = 0.02). **B.** Regression plots showing the relationship between the expression of Nav1.9 and VAS scores for pain, tingling and discomfort as reported by the patient, revealed there was no significant correlation between the degree of symptoms experienced and the level of Nav1.9 expression.

The thirteen neuromas were initially selected from the archive based on the highest and lowest VAS scores for symptoms of dysaesthesia, with no bias given to gender, age or length of time between the initial injury and the subsequent surgical nerve repair. Four of the thirteen specimens were from male patients, and nine were from female patients. Statistical analysis of the pooled male and female data revealed that there was no significant difference between the level of expression of Nav1.8 in male (mean 24.53%) and female (mean 20.86%, *p* = 0.72) specimens, and the same was true for the symptomatic females (31.55%) versus symptomatic males (mean 32.18%, *p* = 0.95). Within the pooled data taken from the female specimens, there was no difference between the levels of Nav1.8 expressed in non-painful neuromas (unpaired *t*-test *p* = 0.08; mean 12.31 ± 5.62 [SEM]%) and painful neuromas (mean 31.55 ± 8.04 [SEM]%). Correlation co-efficient analysis of the data revealed that there was no association between the level of Nav1.8 expression and either the age of the patient or the time between the initial nerve injury and the subsequent surgical nerve repair. However, whilst there was no relationship between the expression of Nav1.8 and the pain or discomfort VAS scores, the level of Nav1.8 expression correlated significantly with the presence of dysaesthesia and reported levels of tingling (Pearson’s Correlation *r* = 0.64, *p* = 0.02) (Figure 3).

### Qualitative and quantitative expression of Nav1.9 in PGP9.5-labelled lingual neuroma tissue

**Figure 4:**
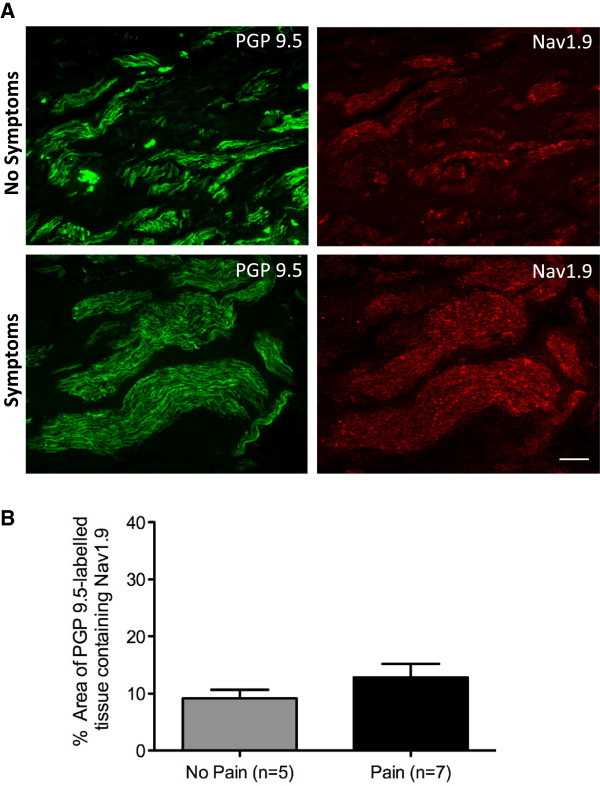
**Nav1.9 is expressed in human lingual nerve neuromas. A.** Nav1.9 immunolabelling appeared greater in lingual nerve neuromas from patients with symptoms of dysaesthesia. Images of lingual nerve neuromas from patients without or with symptoms of dysaesthesia, showing double labelling with the general neuronal marker PGP9.5 (green) and Nav1.9 (red). Scale bar, 100 μm. **B.** The mean percentage area (± SEM) of PGP9.5 labelled tissue containing Nav1.9 did not reveal any significant differences between the two groups of neuromas (unpaired t-test p = 0.27; no pain 9.16 ± 1.51%, pain 12.8 ± 2.38%).

Neuromas processed for PGP9.5 and Nav1.9 showed immunoreactivity to PGP9.5 in all specimens, whilst only twelve of the thirteen specimens showed positive immunoreactivity to Nav1.9. The PGP9.5 labelling was bright and uniform throughout the sections, showing good nerve fibre specificity (Figure 4). The PGP9.5 labelling highlighted the extent of nerve injury and axon disruption, and in many of the specimens the site of the initial injury was clear. In the twelve neuromas that were positively stained for Nav1.9, immunoreactivity was bright and of a pin-prick appearance, showing co-expression with PGP9.5 labelled tissue. However, the level of expression appeared to vary with a qualitative assessment suggesting that Nav1.9 expression was higher in neuromas from patients with symptoms of dysaesthesia (Figure 4). One of the neuroma specimens (specimen 5) showed no Nav1.9 staining, and the appearance of the sections suggested that the Nav1.9 primary antibody may have failed to bind to the tissue, and for this reason was excluded from the quantitative analysis.

The percentage area of PGP9.5 that contained Nav1.9 immunoreactivity varied greatly between the specimens sampled (Table 1), ranging from 5.42-13.39% (mean 9.16 ± 1.51 [SEM]%) in neuromas from patients with no symptoms of dysaesthesia, and 1.12-18.97% (mean 12.8 ± 2.38 [SEM]%) in neuromas from patients with symptoms of dysaesthesia. Statistical analysis revealed that there was no significant difference between these two groups (unpaired *t*-test *p* = 0.27) (Figure 4). Of the twelve specimens expressing Nav1.9, four were from male patients, and eight were from female patients, with no significant difference between Nav1.9 levels in the pooled data from males (mean 9.94%) and females (mean 13.96%, *p* = 0.24). Furthermore, analysis showed that there was no difference in levels of Nav1.9 expression between the non-symptomatic female specimens (mean 8.10%) and the symptomatic female specimens (mean 11.79%, *p* = 0.54). The mean age of the patients with no symptoms of dysaesthesia was 30.33 (± 1.96) years compared with 32 (± 2.92) years for patients with symptoms of dysaesthesia, and correlation co-efficient analysis revealed there was no significant correlation between the level of Nav1.9 expression and the age of the patient. Further correlation coefficient analysis of the data revealed that in both the non-symptomatic and symptomatic groups of neuroma specimens, there was no significant relationship between the level of Nav1.9 expression and the VAS scores for pain, tingling or discomfort, or the time between the initial nerve injury and repair (Figure 3).

## Discussion

Data from this study show that Nav1.8 and Nav1.9 are present in human lingual nerve neuromas, and provide the first quantitative demonstration that expression of Nav1.8 is significantly higher in painful lingual nerve neuromas compared with non-painful neuromas. Furthermore a significant positive correlation between the level of Nav1.8 expression and the degree of tingling experienced by the patients was revealed, suggesting that this sodium channel subtype may have an important role in the development and/or maintenance of neuropathic pain and the symptoms of tingling arising in the lingual nerve after a peripheral injury. However, our data indicate that elevated levels of Nav1.9 may not play a significant role in the generation and maintenance of lingual nerve injury induced neuropathic pain.

Previous studies in our laboratory have investigated the expression of a number of known mediators of neuropathic pain including Nav1.7, P2X3, P2X7, TRPV1, and TRPA1 [22,26-29], and described their presence in human lingual nerve neuroma specimens. Quantitative immunohistochemical protocols revealed that there was no significant difference in expression of any of these mediators, including the sodium channel subtype Nav1.7, in lingual nerve neuromas associated with or without symptoms of dysaesthesia. Furthermore there was no correlation with patient VAS scores for pain, tingling or discomfort, thus suggesting that despite these previously described mediators of neuropathic pain being expressed at the site of injury, elevated levels of expression of these molecules does not appear to play a primary role in the development or maintenance of neuropathic pain arising as a result of lingual nerve injury.

A number of studies have examined the expression of multiple sodium channel subtypes in injured human nerve specimens over the past twenty years. England and colleagues [30] were the first to report an accumulation of sodium channels within the tips of human injured axons, that was significantly higher in painful neuromas compared to control nerves [31]. The idea that such an increased accumulation may play a role in the generation of ectopic axonal excitability has propelled attempts to determine the exact role sodium channels play in the underlying mechanisms of pain, with the aim of developing target specific, low side effect therapeutic treatments. A study conducted by Coward and colleagues [32] used antibodies specific for the individual sodium channel subtypes, and reported a marked increase in the expression of Nav1.8, but not Nav1.9, immunoreactive fibres, in distal limb neuromas from patients experiencing chronic hyperalgesia and allodynia. The authors postulated that the increase in expression of Nav1.8 at the site of nerve injury was due to a translocation of Nav1.8 from the site of production within the DRG, as confirmed by a decrease in Nav1.8-immunoreactive cell bodies. Black et al. [11] reported an upregulation of Nav1.8 immunofluorescence, accompanied by an accumulation of activated p38 and ERK1/2, in painful neuroma tissue when compared with control nerve – a portion of nerve tissue excised proximal to the neuroma and thus acting as its own control. In keeping with our results, and those of Coward and colleagues [32], Black et al. [11] did not see an increase in expression of Nav1.9 immunofluorescence in painful human nerve neuromas. Differences between these studies and the current study relate to the comparison of Nav1.8 and Nav1.9 levels in injured nerve and control nerve. However, the present study has compared differences between injured symptomatic vs. non-symptomatic neuromas, and this enables the study of differences specifically related to the presence of pain – as opposed to differences related to the presence of injury. An increase in Nav1.8 immunoreactivity in painful neuromas compared to non-painful neuromas and control nerves was described by Kretschmer et al. [33], however this was a qualitative study, reporting a pronounced impression, rather than a quantified level. In the present study, we were unable to examine the expression of Nav1.8 and Nav1.9 in human control lingual nerve and trigeminal ganglion tissue, and thus we were unable to determine how sodium channel subtype expression in the lingual nerve neuromas compares to that seen in normal nerves, and whether it correlates with changes in expression in the trigeminal ganglion. However, other studies have shown very low levels of staining for sodium channels in normal peripheral nerves [33,34].

Voltage-gated sodium channels play a key role in the initiation and propagation of action potentials, and it is now widely accepted that changes in sodium channel expression and their accumulation at sites of peripheral nerve injury may contribute to the mechanisms underlying the hyperexcitability that develops in sensory neurones following peripheral nerve axotomy. Expressed preferentially in small diameter sensory neurons in the periphery [4], TTX-r sodium channel subtypes Nav1.8 and 1.9 have electrophysiological characteristics that enable them to remain persistently active [35], and recover from inactivation rapidly [36], thus contributing to hyperexcitability of primary afferents that can bombard the CNS with a stream of action potentials. Studies employing transgenic knockout mice models reported the suppression of both spontaneous activity recorded from the site of nerve injury, and the ability to sense cold pain or mechanical pressure in a Nav1.8 null mouse [37]. Furthermore, Nav1.8 null mice showed reduced pain behaviour and no hyperalgesic response to capsaicin, when compared with their wild type littermates [38]. However these findings are not universal and other studies using Nav1.8 null mice have demonstrated that Nav1.8 is not essential for neuropathic pain behaviour [39]. Lai et al. [40] reported that injection of antisense RNA to Nav1.8 removed all sensitisation to mechanical and thermal stimuli in animals that had undergone a spinal nerve injury, which returned following the removal of the antisense. In animal models of neuropathic pain, transcripts and proteins of Nav1.8 and 1.9 were significantly reduced in the DRG tissue, which was accompanied by a significant attenuation of the currents produced by these channels [41,42]. Consistent with our study and the expression of Nav1.8 and Nav1.9 in human trigeminal nerve tissue, Davies et al. [34] reported an accumulation of Nav1.8 and 1.9 at the site of inferior alveolar nerve transection in the ferret, with a down-regulation of Nav1.8 mRNA in small sized trigeminal ganglion cells following inferior alveolar nerve injury in the rat [43].

Transgenic mouse models and loss of function studies suggest that different voltage-gated sodium channel subtypes are associated with different types of pain (for review see [5]). Responsible for the persistent TTX-r current in small diameter sensory neurons [44], strong evidence now exists that Nav1.9 plays a major role in mediating inflammatory rather than neuropathic pain, and this may explain our data and the absence of a significant difference in the levels of Nav1.9 expression in lingual nerve neuromas with and without symptoms of pain. A knock-out mouse study by Maingret and colleagues [45] reported the failure of inflammatory mediators bradykinin, ATP, histamine, prostaglandin-E2 and norepinephrine, to sensitise sensory neurons in Nav1.9 null mice. In contrast, wild type mice were able to upregulate Nav1.9 channel activity when an inflammatory soup was applied, thus suggesting that Nav1.9 is a crucial player in inflammation, contributing to nociceptive hyperexcitability. Studies examining the expression of Nav1.8 and Nav1.9 in other chronic inflammatory pain models have reported an increased expression in painful inflamed human dental pulp tissue, when compared with normal non-painful pulps [46-48], and in a Freund’s complete adjuvant (FCA) model of chronic inflammatory joint pain, Nav1.8 and Nav1.9 expression in associated DRG cells was upregulated, up to 28 days post-FCA insult [49].

It might have been expected that the length of time between the initial injury and the subsequent repair surgery would affect the level of expression of Nav1.8 or Nav1.9, or the VAS scores associated with the symptoms experienced by the patients; however, this was not the case. A time course study of sodium channel subtype expression in animal models of neuropathic pain reported that after initial alterations in sodium channel expression and accumulation, changes revert to normal relatively quickly [43]. However in the case of the human nerve study, neuroma specimens were obtained after a relatively long period of time after the initial injury and so the early changes seen in the animal studies may not have been detected. Indeed it might have been expected that sodium channel expression would have reduced to normal levels in our specimens, but this did not appear to be the case with marked variation across the whole set of specimens, and the lack of any normal control tissue makes interpretation of this variation difficult.

## Conclusions

In summary, our data have shown that Nav1.8 and Nav1.9 are expressed in human lingual nerve neuromas. Despite the level of expression showing wide variation between the specimens sampled, results from this study provide the first demonstration of a statistically significant difference in the levels of Nav1.8 in lingual nerve neuromas from patients experiencing painful symptoms of dysaesthesia, compared with those from patients experiencing no symptoms of dysaesthesia. Furthermore, the level of expression of Nav1.8 correlated significantly and positively with reported levels of tingling (VAS) as reported by the patient. Elevated expression of Nav1.8 appears to be important in the development and/or maintenance of injury-induced neuropathic pain in the lingual nerve.

## Methods

### Neuroma specimens

Ethical approval for the study was obtained from the South Sheffield Research Ethics Committee, and all the specimens were collected with the patients’ informed consent. All patient details were kept confidential and each specimen was given a unique code to be used throughout the study. Specimens were obtained from our archive of 84 lingual nerve neuromas collected from patients referred to the Charles Clifford Dental Hospital Oral and Maxillofacial Medicine and Surgery Department, from throughout the United Kingdom, between 2000 and 2008. The light and microscopic characteristics of these neuromas has been described previously [50]. For a detailed description of the surgical removal and repair of the lingual nerve, see [51]. Clinical histories and symptoms were obtained pre-operatively using a questionnaire, and patients asked whether the affected part of the tongue was painful, and whether they had any tingling either spontaneously or caused by moving or touching their tongue. Furthermore, patients scored the extent of their pain, tingling and discomfort using visual analogue scales (VAS). These tests revealed that patients suffered either anaesthesia or a significant degree of hypoaesthesia, in addition to variable degrees of dysaesthesia, on the side of the tongue innervated by the damaged nerve, thus confirming the need for further exploration and surgical repair of the nerve. Thirteen 'neuroma-in-continuity’ specimens were selected from the archive. These neuromas all had some element of nerve tissue bridging the gap between the central and distal stumps of the damaged nerve. Seven specimens were categorized as from patients with the most severe symptoms, where patients experienced either pain or unpleasant tingling either spontaneously or initiated by touching or moving their tongue. The other 6 specimens were categorized as from patients with no symptoms of dysaesthesia, as there was a complete absence of pain or unpleasant tingling reported at the time of clinical examination and pain questionnaire.

### Tissue processing

Immediately following surgical removal of the neuroma, the central end was marked with a 9/0 Ethilon (Ethicon, Edinburgh, UK) suture and the specimen placed in 2% Zamboni’s fixative (0.1 mol/L phosphate buffer, pH 7.4, containing 4% paraformaldehyde and 0.2% picric acid) for 24 hours at 4°C. Following fixation, the specimen was cryoprotected in 30% sucrose solution for 12 hours at 4°C, and embedded longitudinally in Tissue-Tek® O.C.T. compound (Sakura Finetek, Europe). Serial 14-μm sections were cut on a microtome cryostat, and thaw–mounted onto poly-D –lysine (Sigma Aldrich Company Ltd, Gillingham, UK) coated glass microscope slides. Sections were collected as 20 sets so that each section was 280 μm from the section adjacent in the same set. Sections were left to air dry for 1 hour at room temperature, prior to storage at -80°C until required for processing.

### Immunofluorescence

Two sets of slides from each specimen were processed for indirect immunofluorescence, and dual labelled with primary affinity-purified antisera to the general neuronal marker protein gene product 9.5 (PGP9.5) and either sodium channel subtype Nav1.8 or Nav1.9 [11,52]. Prior to staining, the slides were removed from storage and left to air dry for 1 hour at room temperature. After washing in phosphate-buffered saline (PBS) (2 × 10 minutes), the sections were incubated in PBS containing 0.5% Triton X-100 (PBST) and 20% normal donkey serum (NDS) for 1 hour at room temperature in a moisture chamber, to reduce non-specific background staining and increase the permeability of cell membranes to the antibodies. The sections were incubated with primary antibodies raised in rabbit to either sodium channel Nav1.8 (1:200) or Nav1.9 (1:2000), diluted in PBST and 5% NDS for 24 hours at 4°C. They were then washed in PBS (2 × 10 minutes) and incubated for 90 minutes at room temperature with secondary antibody raised in donkey against rabbit IgG, conjugated to indocarbocyanine (Cy3, Jackson ImmunoResearch Laboratories Inc, USA, 1:500), diluted in PBST containing 1.5% NDS. The tissue was further washed in PBS (2 × 10 minutes), and then incubated with a monoclonal primary antibody raised in mouse against human PGP9.5 overnight at 4°C (UltraClone, 1:1000; diluent: PBST 5% NDS). The following day the sections were washed in PBS (2 × 10 minutes) and incubated with a fluorescent secondary antibody, raised in donkey against mouse IgG, conjugated to fluorescein isothiocyanate (FITC; Jackson, 1:50, diluted in PBST containing 1.5% NDS) for 90 minutes at room temperature. The sections were washed one final time in PBS (2 × 10 minutes), mounted in fluorescence-free Vectashield medium (Vector Laboratories, UK) and coverslipped.

Immunohistochemical controls for PGP9.5, Nav1.8 and Nav1.9 were performed by incubating the tissue sections with the secondary antibody alone.

### Examination and analysis of tissue

Images of sections were acquired with a Zeiss Axioplan 2 Imaging fluorescence microscope, fitted with a HBO 50 mercury lamp, ×10 eyepiece and excitation/emission filter sets for FITC and Cy3 detection. Image acquisition and processing was performed using Image-Pro Plus (v5.1, Media Cybernetics, USA). Analysis was performed blinded to the specimen group.

A section from approximately the middle of each specimen was selected for analysis. In order to determine the proportion of nerve tissue that contained the specific sodium channel, the percentage area of PGP9.5 labelled tissue, which was also labelled for the specific sodium channel, was quantified. For each specimen, the section that contained the most PGP9.5 labelled tissue was selected for analysis. Preliminary assessments revealed that analysis of 150,000 μm^2^ of PGP9.5 labelled tissue provided an accurate representation of the whole section. Sampling was undertaken across the full width of the neuroma, starting from the central cut end of the nerve, and extending along the nerve for 3 fields of view (×20 objective), or until at least 150,000 μm^2^ of PGP9.5, had been analysed.

Initially each field of interest was viewed with the FITC filter and the area of PGP9.5 labelling measured. The same field was then viewed with the Cy3 filter in place and the area of each sodium channel subtype staining measured. The total area of PGP9.5 and sodium channel staining within the area analysed was measured and the total percentage area of PGP9.5 labelled tissue, which was also positively stained for the sodium channel calculated.

### Statistical analysis

Statistical analysis was performed using GraphPad Prism (Version 5.0d, GraphPad Software, Inc. La Jolla, CA, USA). Unpaired sample *t-*tests were employed to compare the levels of expression of Nav1.8 and Nav1.9 in non-symptomatic neuroma specimens with symptomatic neuromas, for differences between neuromas from male and female patients, and between neuromas from females with and without symptoms. Differences were considered statistically significant if *p* < 0.05. Pearson’s correlation coefficients were used to establish any correlations between the expression of Nav1.8 and Nav1.9 and the VAS scores for pain, tingling, or discomfort; the age of the patient; or the time between the initial nerve injury and repair.

## Competing interests

The authors declare no competing interests.

## Authors’ contributions

FMB, PPR and EVB together conceived, initiated and designed the study. EVB and CRC were responsible for undertaking the experiments and analysis, while FMB oversaw the project. All clinical data were collected, and surgical excision of the lingual nerve neuromas carried out by oral and maxillofacial surgeons PPR and KGS. The sodium channel antibodies were developed and validated by JAB and SGW. ARL was responsible for gaining and maintaining the ethical approval required for the project. EVB wrote the manuscript with input from CRC, JAB, SGW and FMB. All authors read and approved the final manuscript.
